# Identifying and characterising key alternative splicing events in Drosophila development

**DOI:** 10.1186/s12864-015-1674-2

**Published:** 2015-08-16

**Authors:** Jonathan G. Lees, Juan A. Ranea, Christine A. Orengo

**Affiliations:** Institute of Structural and Molecular Biology, Division of Biosciences, University College London, Gower Street, London, WC1E 6BT UK; Department of Molecular Biology and Biochemistry–CIBER de Enfermedades Raras, University of Malaga, Malaga, 29071 Spain

## Abstract

**Background:**

In complex Metazoans a given gene frequently codes for multiple protein isoforms, through processes such as alternative splicing. Large scale functional annotation of these isoforms is a key challenge for functional genomics. This annotation gap is increasing with the large numbers of multi transcript genes being identified by technologies such as RNASeq. Furthermore attempts to characterise the functions of splicing in an organism are complicated by the difficulty in distinguishing functional isoforms from those produced by splicing errors or transcription noise. Tools to help prioritise candidate isoforms for testing are largely absent.

**Results:**

In this study we implement a **T**ime-course **S**witch (TS) score for ranking isoforms by their likelihood of producing additional functions based on their developmental expression profiles, as reported by modENCODE. The TS score allows us to better investigate functional roles of different isoforms expressed in multi transcript genes. From this analysis, we find that isoforms with high TS scores have sequence feature changes consistent with more deterministic splicing and functional changes and tend to gain domains or whole exons which could carry additional functions. Furthermore these functions appear to be particularly important for essential regulatory roles, establishing functional isoform switching as key for regulatory processes. Based on the TS score we develop a Transcript Annotations Pipeline for Alternative Splicing (TAPAS) that identifies functional neighbourhoods of potentially interesting isoforms.

**Conclusions:**

We have identified a subset of protein isoforms which appear to have high functional significance, particularly in regulation. This has been made possible through the development of novel methods that make use of transcript expression profiles.

The methods and analyses we present here represent important first steps in the development of tools to address the near complete lack of isoform specific function annotation. In turn the tools allow us to better characterise the regulatory functions of alternative splicing in more detail.

**Electronic supplementary material:**

The online version of this article (doi:10.1186/s12864-015-1674-2) contains supplementary material, which is available to authorized users.

## Background

Multicellular organisms must robustly carry out complex developmental and regulatory tasks with a limited set of genes. Indeed across the range from simple to complex organisms, (e.g. as defined by the number of cell types), there are a surprisingly similar number of genes. Why gene number is a poor predictor of organism complexity was initially referred to as the G-value paradox [1]. This apparent paradox was used to point out that the actual ‘Information value’ of a genome (or I-value) could be explained by many other sources such as increased gene regulation, protein interactions, multi-functional genes or total proteome size [2].

Increasing the complexity of a genes expression profile through cis regulation, could also in principal increase its number of functions [3].

Another source of potential functional expansion comes from the now common observation of single genes encoding multiple transcript isoforms. Many of these isoforms have different protein sequences, producing a potential expanded isoformal proteome. Furthermore, specific transcript isoforms are thought to be important for gene regulation (e.g. [4]) with roles in human diseases such as cancer [5] and autism [6]. Domain based annotations have shown alternative splicing in general to be associated with cell communication, signalling development and apoptosis [7].

A major process generating transcript diversity from a single gene is through alternative splicing (AS), whereby amongst other mechanisms variable inclusion of exons and altered exon boundaries can be used to produce different transcripts. Transcript isoforms can also be generated from alternative transcript initiation (ATI) and termination (ATT). An example of this is seen for the TP73 gene where proteins with opposing functions are generated [8]. The number of isoforms generated from ATT and ATI appears to be extremely large [9, 10] and in combination with splicing can produce a myriad of isoform patterns requiring a more complete set of definitions to fully describe them [11].

It has been shown that a multi transcript gene typically has a major transcript isoform accounting for the majority of the expression. The other minor transcripts from a gene are generally expressed at much lower levels and restricted to fewer cell types [12]. It is important to note that a genes transcript isoforms often code for identical protein coding sequence, and only alter their 3’ or 5’ untranslated regions (UTRs) although this can also have functional consequences (Table 1), in this paper we focus on transcripts which change their protein coding sequence.

**Table 1:** Consequences of Alternative splicing. Location of transcript diversity and examples of functional consequences

Region altered in transcript	Example functions associated with Change
ORF	Protein interactions, Enzymatic activity, stability
5’ UTR	Expression level, initiation or degradation rates, scanning efficiency, Ribosome procession, stability
3’ UTR	Sub-cellular localisation initiation, degradation, stability

For functionally annotating genes various databases exist that gather information on genes, or link genes together allowing the functional neighbourhood of a gene to be investigated. For multi protein genes, isoform level annotation is not possible since the annotations are only rarely isoform specific in sequence annotation databases (GO [13], INTACT [14] etc.).

In a number of cases changes in the sequence features, such as gain/loss of a globular domain, transmembrane anchor or signal peptide allow some high level functional interpretation. In Swiss-Prot, 36 % of the isoforms alter the domain architecture of the proteins [15] and these switch events are amenable to function annotation methods such as the InterPro [16] or FunFam pipelines [17]. For the remaining sequences other non-domain based approaches are required. Protein function prediction challenges such as CAFA [18], where teams compete to predict future GO assignments for proteins have shown the utility of machine learning approaches for gene level function prediction. However, the absence of isoform specific function annotation severely limits the usefulness of machine learning approaches for isoform function prediction.

Despite the great abundance and variety of isoforms produced in complex organisms, demonstration of the global functional importance of much of this remains to be established and it is an open question as to how much is functionally adaptive and how much is noise from the splicing process [19, 20]. Furthermore, a significant proportion of the isoforms produced may code for unstable and potentially harmful proteins. For example, recent analysis of proteins isoforms predicted from the ENCODE data suggested that functional proteins were “the exception rather than the rule” [21] and that many of the isoforms are simply tolerated. Furthermore the authors quote “Exhaustive literature searches on the genes in this data set unearthed very little evidence of an increase in protein function repertoire” for alternative splicing. It was also noted in this paper that at present researchers can “do little more than hypothesize as to the functional importance of splicing events”. Hence, any method that could systematically detect isoform candidates for functional testing would be a useful research tool.

**Fig. 1:**
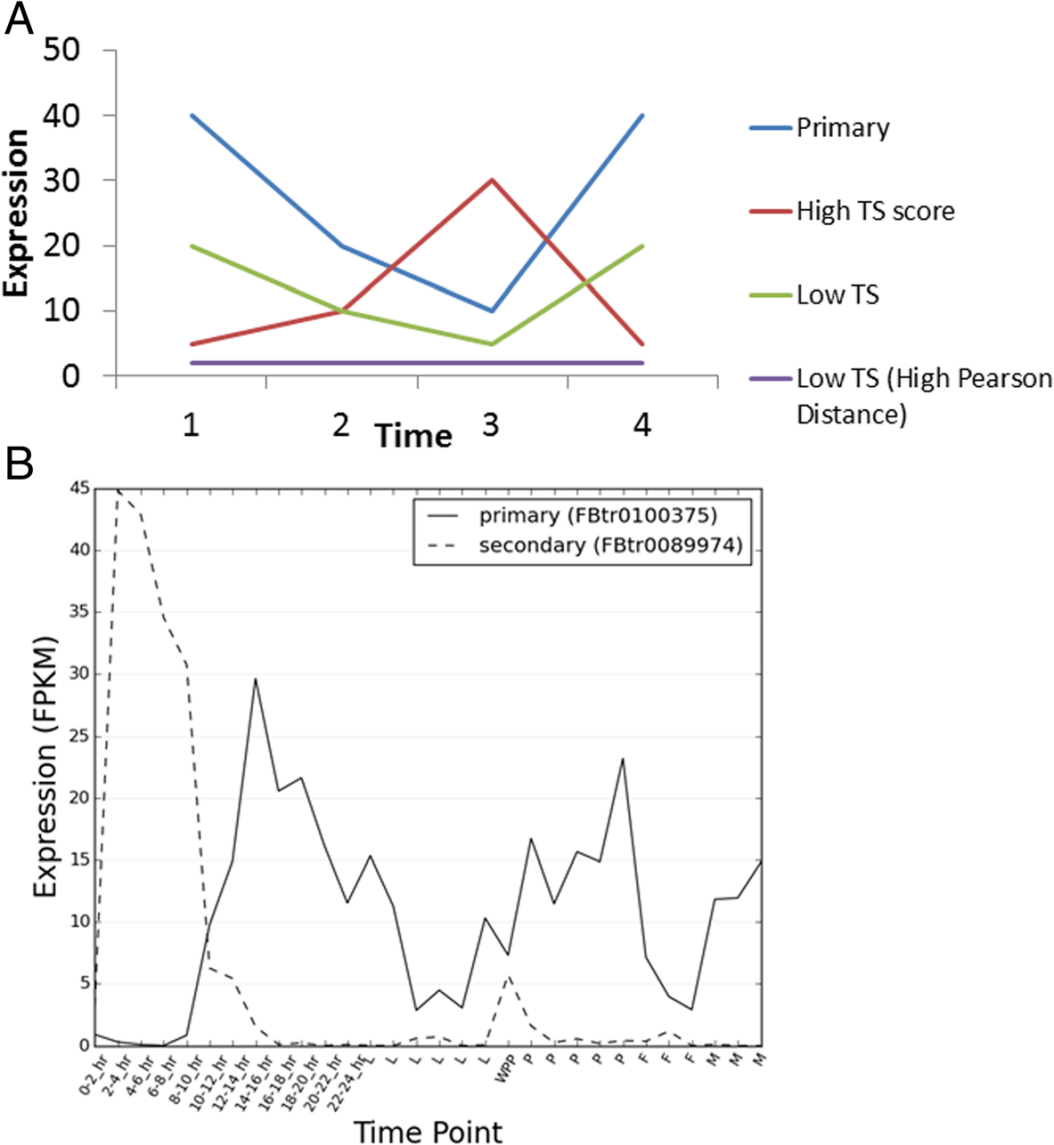
TS score explanation. **a** Hypothetical examples of time course transcript expression profiles from a gene to illustrate the concept of the TS score. The primary transcript is shown in blue. Minor transcripts are shown in other colours. The red line would have a high TS score since it has reasonably high expression relative to the primary transcript but with a different shape. The green transcript would have a Low TS score since although it has high general expression it has the same shape as the primary transcript. The purple transcript would have a low TS score since although it has a different shape it has low expression relative to the primary transcript. **b**, Example of a secondary isoform taken from the ‘sdt’ gene with a high TS score when compared to the primary isoform. The legend IDs between brackets are FlyBase transcript ID’s. Along the x-axis the first 24 hours correspond to embryogenesis stages; time points labelled ‘L’ correspond to subsequent Larval developmental stages; WPP indicates the **w**hite **p**re**p**upae and,‘P’ indicates pupal stage, ‘F’ and ‘M’ are for female and male adult stages respectively (see Additional file 1: Figure S1 for an example of a gene with a low TS score)

In this article we develop tools for bridging this gap and allowing more detailed functional analysis of splicing. We develop a pipeline that identifies alternative splice variants with significantly different expression profiles. Here we make use of isoform expression profiles over a developmental time series in D.melanogaster [22] to identify alternative protein isoforms that have expression profiles indicative of function. The idea of gene expression divergence corresponding to functional divergence is often found in the literature (for example [23]), and in this study we adapt these ideas for comparing intra gene expression profiles rather than inter gene. The score we developed for this we name the **T**ime-course **S**witch (TS) score (see Eq. 1 and Fig. 1a) which takes into account both the magnitude and expression profile differences between primary and minor transcript expression profiles in its calculation.

Ultimately, the tools we present here help us to better characterise the roles of alternative splicing in development and we find that within the set of genes that undergo alternative splicing, those with high TS scores are enriched in regulatory processes. We develop a novel algorithm TAPAS enabling the functional neighbourhood of isoforms with high TS scores to be investigated.

## Results

### Measures of isoforms expression variation and datasets classification

We obtained the paired end sequence data from the fly developmental modENCODE project and calculated expression levels of isoforms from FlyBase 5.45 for all developmental stages using the same protocol as used in the original modENCODE analysis (see Methods).

From the outset we restricted our analysis to multi protein genes, that code for multiple protein isoforms and where each isoform shows a minimal expression of 1 FPKM (see Table 2) in at least one of the developmental stages of the modENCODE data.

**Table 2:** Glossary of Terms. Glossary of common terms and their definitions used in this article

Term	Description
High-TS genes	We identified isoforms with high TS scores (>0.5) and refer to them as High-TS genes. A value of 0.5 was chosen since this gave significant shape differences on manual visual inspection whilst maintaining a sufficiently large number of multi protein genes for statistical tests.
Primary isoform	The transcript of a gene with the maximum average expression.
Secondary isoform	The transcript of a gene with a different protein sequence to the primary isoform and with the next highest average expression level.
Minor isoform	Any transcript with a different protein sequence to the primary isoform.
Multi protein gene	A gene coding for at least two different protein isoforms. In this study we also filtered to only include isoforms if they were expressed >1FPKM in at least one developmental stage.
Intron Retention (IR)	Intron retention occurs when the intron of gene fails to be removed from the between neighbouring exons.
Exon Gain/Loss	Splicing events where a whole exon is gained or lost in one transcript relative to another.
Conservation Index (CI)	The conservation index as defined in the modENCODE validation paper measures the evolutionary distance at which a genomic element (e.g. exon) can be identified as expressed by RNAseq data. Greater values indicate greater evolutionary distances.
FPKM	FPKM (Fragments per kilobase of exon per million reads mapped) is a standard measure of expression for RNAseq data.

From the modENCODE dataset for each multi protein gene, we ranked in decreasing order, each of the transcripts by their mean expression levels over the developmental time course in Drosophila. For each gene we identified the primary transcript as that with the highest average expression. We refer to the second most highly expressed transcript with a different protein sequence to the primary transcript as the secondary transcript. For multi protein genes we found that 77 % of the genes overall expression was from the primary transcript, increasing to 89 % if the secondary isoform was included. Hence, it is observed that the interchange between the primary and the secondary isoform represents a dominant switching event in multi protein genes.

We sought a simple metric obtainable from a minor isoform’s expression pattern that could be used as an indicator of function. At one extreme, if a minor isoform is expressed at very low levels compared to the primary one and at the same time and location, we might expect a reduced chance of it providing extra functions. Conversely if a minor isoform is expressed at comparable levels to the primary one and at times or locations that the primary isoform is absent or reduced, then this could be a useful indicator of some additional functional roles. The score we developed for this we named the **T**ime-course **S**witch (TS) score (see Eq. 1 and Fig. 1a) which takes into account both the relative magnitude and expression profile differences between primary and minor transcript expression profiles in its calculation. The TS score has the desirable property that it gets higher as the primary and secondary isoforms expression profiles becomes both more dissimilar in shape, and more equal in overall expression (Additional file 1: Figure S2).

$$ TS=2\times \left(1-\frac{s_1}{sum(s)}\right) $$

*Equation 1: The TS score calculation is straightforward using Single Value Decomposition (SVD), where s is a vector corresponding to the singular values of matrix X and s1 is the largest singular value. X is simply a matrix with two row vectors consisting of the primary and secondary transcripts expression profiles.*

The TS score is more useful for our current study than the Pearson similarity measures which is dominated by shape differences. For example the minor transcript represented by the purple line in Fig. 1, is quite flat and lowly expressed, yet would have a large dissimilarity to the primary transcript as measured by the Pearson score (where dissimilarity is measured as 1-Pearson correlation) and so if this scoring method was used for target prioritisation (instead of the TS score) then this minor isoform would be ranked highly even though it could potentially just be noise. We have checked against other distance measures (Additional file 1: Figure S3) and find that the TS score compares favourably, at least for the current problem.

The distribution of TS scores (Additional file 1: Figure S4) shows that 16 % (486 cases) of the multi protein genes have a high TS score (>0.5) between their primary and secondary isoforms. The biological significance of an isoform having a high TS score could correspond to it having a higher likelihood of an important function for the organism.

### Higher TS scores correspond to general characteristics of functional gain

Studying isoform specific functions is complicated since databases rarely have isoform specific information. For example although the high quality INTACT [14] database is systematic in its isoform annotation, the isoformal interactome makes up only a tiny proportion of all its interactions. Similarly, database annotations such as those from the Gene Ontology [13] are almost exclusively at the gene level for *D. melanogaster*. Hence in the analysis of the functional implications of the changes between isoforms we are limited to using proxies of function to identify potential changes.

**Fig. 2:**
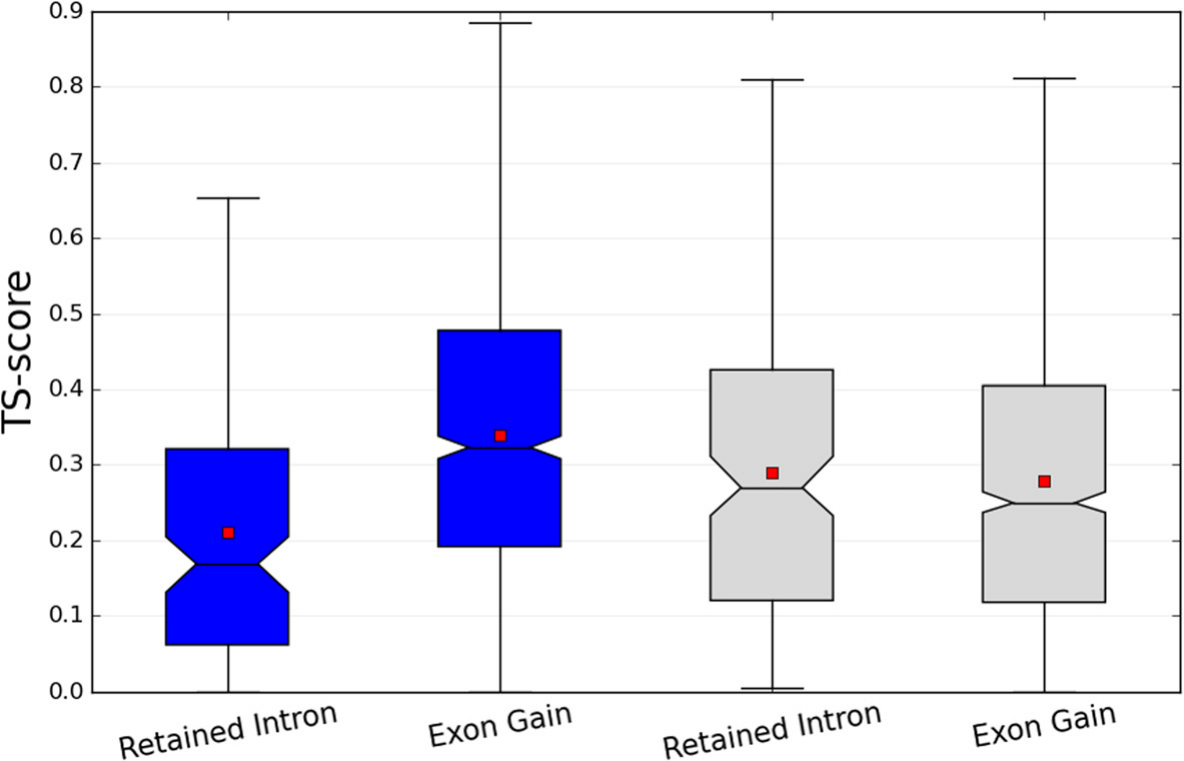
TS scores for different splicing events. TS score distribution of retained intron and exon gain events for real (blue bars), and randomised (grey bars) data. The randomised scores were generated by randomly permuting the TS scores between genes. The box extends from the lower to upper quartile values of the data, with a line at the median. All values more than 1.5 IQR lower than the first quartile or 1.5 IQR higher than the third quartile are excluded as outliers for visualization purposes. The smallest and highest values that are not outliers are connected with a line. The notches correspond to 95 % confidence interval for the median

The gain of exons is known to potentially alter the precise functioning of a protein isoform by changing its interaction partners if certain sequence features are present [24]. Conversely, in the literature Intron retention (IR) is more often perceived as an aberrant, mis-splicing event compared to whole exon gain or loss [25]. For this reason we decided to assess IR events in relation to the TS score to see if we could see any differences from the background rate. We find that exon gains and intron retentions have significantly higher and lower TS scores respectively than expected by chance (Fig. 2), both with Mann–Whitney p-values <0.001. This feature was also observed if only evolutionarily conserved isoform specific exons were considered (see methods). This observation is important since, evolutionary conserved exons are more likely to be functional than those with low evolutionary conservation. Evolutionary conserved exons were detected using the Conservation Index score [26] (Table 2) which measures the evolutionary distance at which an exon is both genomically conserved and shows signs of expression.

### High TS score isoforms gain functional sequence features

#### Functional motifs mediating protein interactions

We next were interested to see if we could identify other protein features with relationships to function in the secondary isoform specific regions. First we assessed if the secondary isoforms of High-TS Genes (those with TS scores >0.5, see Table 2) were more likely to gain a ligand binding Eukaryotic Linear Motif (LIG-ELM) in their secondary isoforms which was absent from the primary isoform (see Methods). LIG-ELMs are short regions of proteins that can mediate protein interactions [27, 28] carrying out regulatory roles. We found there was a significant enrichment for High-TS Genes to have a LIG-ELM in their secondary isoform not present in their primary isoform (Fisher’s p-value = 2.4^−7^). This indicates that functional expansions may be obtained from ELM gain events. As an example, the LIG-NRBOX motif, which is known to have roles in development, is significantly more likely to be gained in secondary isoforms of High-TS Genes (Fisher’s p-value = 2.3^−5^) compared to other multi protein genes.

#### Domains associated with novel functions

**Fig. 3:**
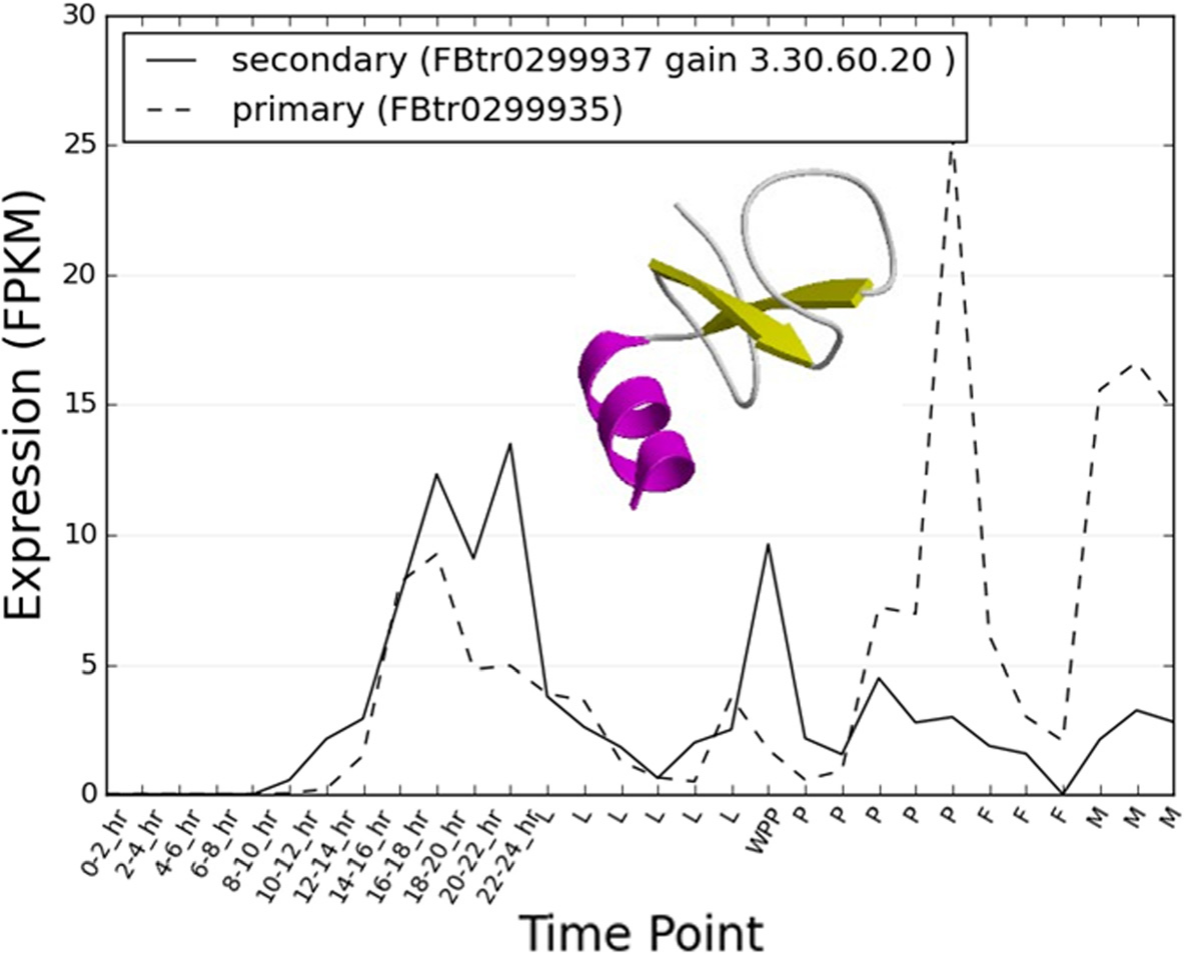
*Domain Switching example.* Example of a domain (functional) gain on switching from primary to secondary isoforms of a High-TS gene. A structural representative of the gained domain family is displayed (ID. in CATH database: 3.30.1370.50). Expression profiles of the transcripts are shown (see Fig. 1b for explanation of axes)

Because of their short sequences, ELMs cannot be assigned with high confidence. However, domains can be assigned with much higher confidence and the mapping of protein domain-family to function is well established. Hence, we can use the domain contents of proteins to detect domain differences between the primary and secondary isoforms to predict any functional changes. In order to obtain high coverage of domain assignments to the protein sequences coded by the isoforms, we made use of the extensive domain annotations in Gene3D [29] a resource that integrates domain assignments from CATH [17], Pfam-A (and Pfam-B) [30] and Superfamily [31]. We found that High-TS Genes were significantly more likely to have an additional domain family in their secondary protein isoform not present in the primary isoform (Fisher’s p-value < 0.01). As an example we can see that for the gene Protein kinase C δ, there is a switch in the maximally expressed isoform at the end of embryogenesis and at the WPP stage to a shorter protein isoform, but which contains an extra domain family (CATH superfamily: 3.30.60.20, C1 domain) (Fig. 3). This domain is thought to be important for the regulation of the kinase through ligand binding [32]. Unlike previous studies which considered all minor isoforms, analyses using our FunFam pipeline (described in methods) did not detect any significantly enriched functions associated with the domains being gained (after correcting for multiple testing using the Benjamini-Hochberg method).

### High-TS Genes are enriched for regulatory functions

We next investigated what functions were associated with High-TS Genes. Functional annotations such as those in the Gene Ontology (GO), protein interaction and RNAi knockdown [33] databases are almost exclusively defined at the gene level. Hence we need to take an indirect approach to assessing the potential functional roles of the high TS scoring isoforms by analysing them at the gene level.

#### Analysing enrichment at the gene level

For function enrichment analysis we tried 2 approaches. Firstly we ran a threshold free GO enrichment approach, GORILLA that accepts a ranked list of genes and looks for enrichment of GO terms near the ‘top’ of the list [34]. Methods such as GORILLA do not require any predefined threshold, so we could simply provide the list of genes ranked by decreasing TS score. The top most significantly enriched terms (q-value < 0.0001) were the general terms (GO:0050896) response to stimulus, (GO:0050794) regulation of cellular process and (GO:0050789) regulation of biological process.

**Fig. 4:**
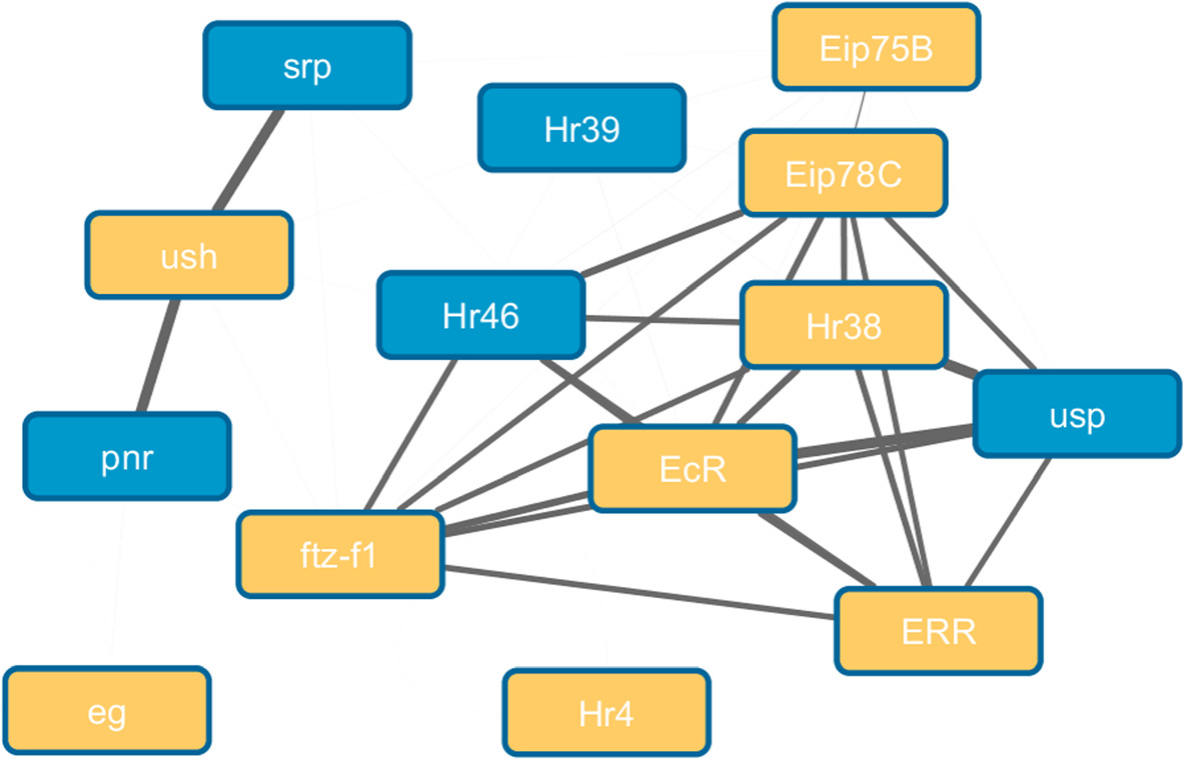
*Example enriched function of High-TS Genes.* Interactions between High-TS Genes annotated with the GO term “direct ligand regulated sequence-specific DNA binding transcription factor activity” (golden nodes). Predicted functionally related genes (predicted using Fun-L [43], see methods) are shown as blue nodes to help provide a more connected network

Because of the lack of specific terms produced by this, we ran a second approach using Fisher’s exact test to look for GO terms enriched in the High-TS Genes compared to the multi protein genes as the background set. The most significantly enriched GO term for High-TS Genes was for “direct ligand regulated sequence-specific DNA binding transcription factor activity” (corrected p-value = 0.025). A narrow synonym given for this GO term in the QuickGo website is “nuclear hormone receptor”. We could see that this set includes many different nuclear hormone receptors that on ligand activation act as on-off switches for the genes they regulate when bound to DNA in a sequence specific manner (Fig. 4).

The nuclear hormone receptors are known to be important in embryonic development and thus our results suggest that dynamic expression switching of the isoforms for many of these genes, is important in this process. We are able to confirm this in the literature for one of these genes, the EcR gene. Differences in the EcR protein isoforms N-terminal regions [35] and expression patterns have been suggested to provide isoform specific functions [35]. Experiments have shown that EcR isoform specific mutants produce lethality at characteristic stages of development [36, 37].

#### Analysing enrichment at the domain level

**Fig. 5:**
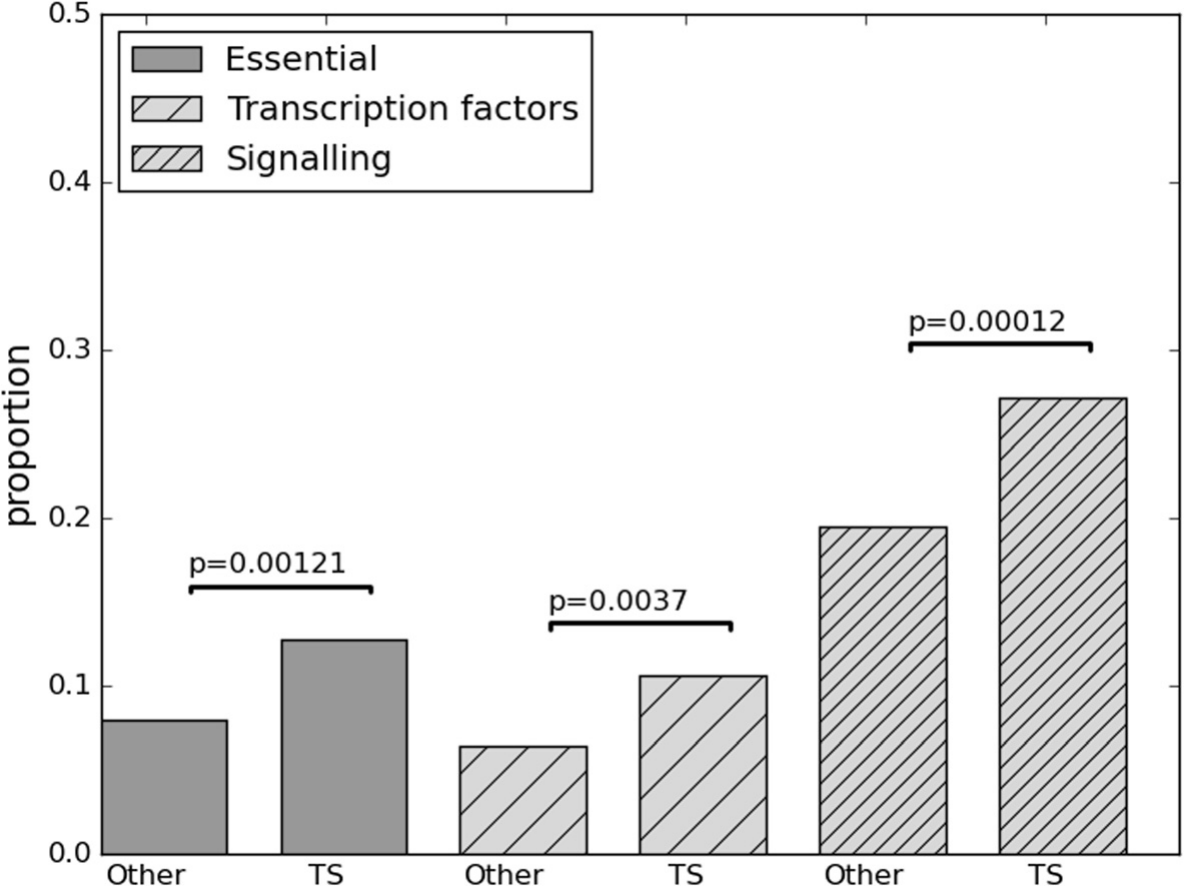
*Other characteristics of High-TS Genes.* Proportions of High-TS Genes, labelled ‘TS’, and all other multi-protein genes, labelled ‘Other’, which are essential, contain transcription factors domains or classical signalling domains (see methods). Lines and values connecting bars indicate Fisher’s exact test enrichment significance

We can also use protein domain assignments in a different way to that used previously. In earlier sections we used the domains to establish specific changes between isoforms, whilst here we can use all the domain assignments of a gene to show the genes overall general function. For certain domain types such as sequence specific DNA binding domains, the functions they carry out can be assigned with high confidence (DBD [38]). When we performed functional assignments to genes in this way, using the DBD [38], we found that there was a significant enrichment in transcription factor/sequence specific DNA binding domains for the High-TS Genes relative to other multi protein genes (Fig. 5). We also found enrichment for proteins containing domains involved in signalling [39].

Hence, the genes containing isoforms with high TS scores are particularly related to regulatory processes involved in organismal development and complexity. For example transcription factors (TF’s) are key drivers of development, important in patterning, segmentation and tissue differentiation. In other work [24] examining human tissue expression, the dynamics of splicing has been identified as potentially important in regulatory processes and despite major differences in approaches our work further supports this idea (see discussion).

### Genes with high TS scores are enriched for essential genes

Databases such as OGEE estimate the age of a gene, by finding the deepest branch in the tree of life in which it appears to have emerged using sequence based methods [40]. We found that the High-TS Genes were significantly enriched in Metazoa genes (Fisher’s p-value = 0.002) and depleted in young genes (Fisher’s p-value = 1.7-5). This indicates that the High-TS Genes tend to originate from a time when complex organisms were first appearing. The High-TS Genes also tend not to be duplicated genes (Fisher’s p-value = 0.0014), i.e. they are enriched in non-duplicated, singleton genes. It has been shown previously that across multiple organisms, older non-duplicated genes are indicators of gene essentiality [41]. To further confirm this potential essentiality enrichment we downloaded the set of essential genes identified from experiments in the OGEE resource (see Methods). We found the High-TS Genes to be significantly enriched in essential genes (Fisher’s p-value = 0.001) (Fig. 5).

It is possible that the gene essentiality may positively correlate with expression features and various protein network properties [42]. However the High-TS Genes were not significantly different in their number of protein interactions or gene expression levels. We also removed any further potential biases in gene expression using a Euclidean based similarity normalisation approach (see methods) and found that the significant enrichment in essential genes remained after this bias correction.

We also checked if the enrichment in essential genes could be explained by other factors (such as the High-TS genes enrichment in signalling, DNA binding or singleton genes), however the trends and significant enrichments remained after controlling for these effects, too (see methods). If as outlined above, the High-TS Genes provide additional functions through their secondary isoforms, we might expect the gene as a whole to be more essential, since knock down of genes that carry out multiple tasks by utilising different transcripts at different times, would remove multiple functions.

### Identification of protein isoform specific functional neighbourhoods

Having developed our TS method for identifying functional splicing events, we sought to develop an algorithm that could provide functional characterisation of isoforms with high TS scores. As mentioned earlier the level of functional annotation of isoforms in existing databases is too low to be useful, making this task particularly challenging. As highlighted in Fig. 1, the TS score will prioritise minor isoforms with an appreciable level of expression to the primary isoform, but where they have differences in their expression profile shape.

**Fig. 6:**
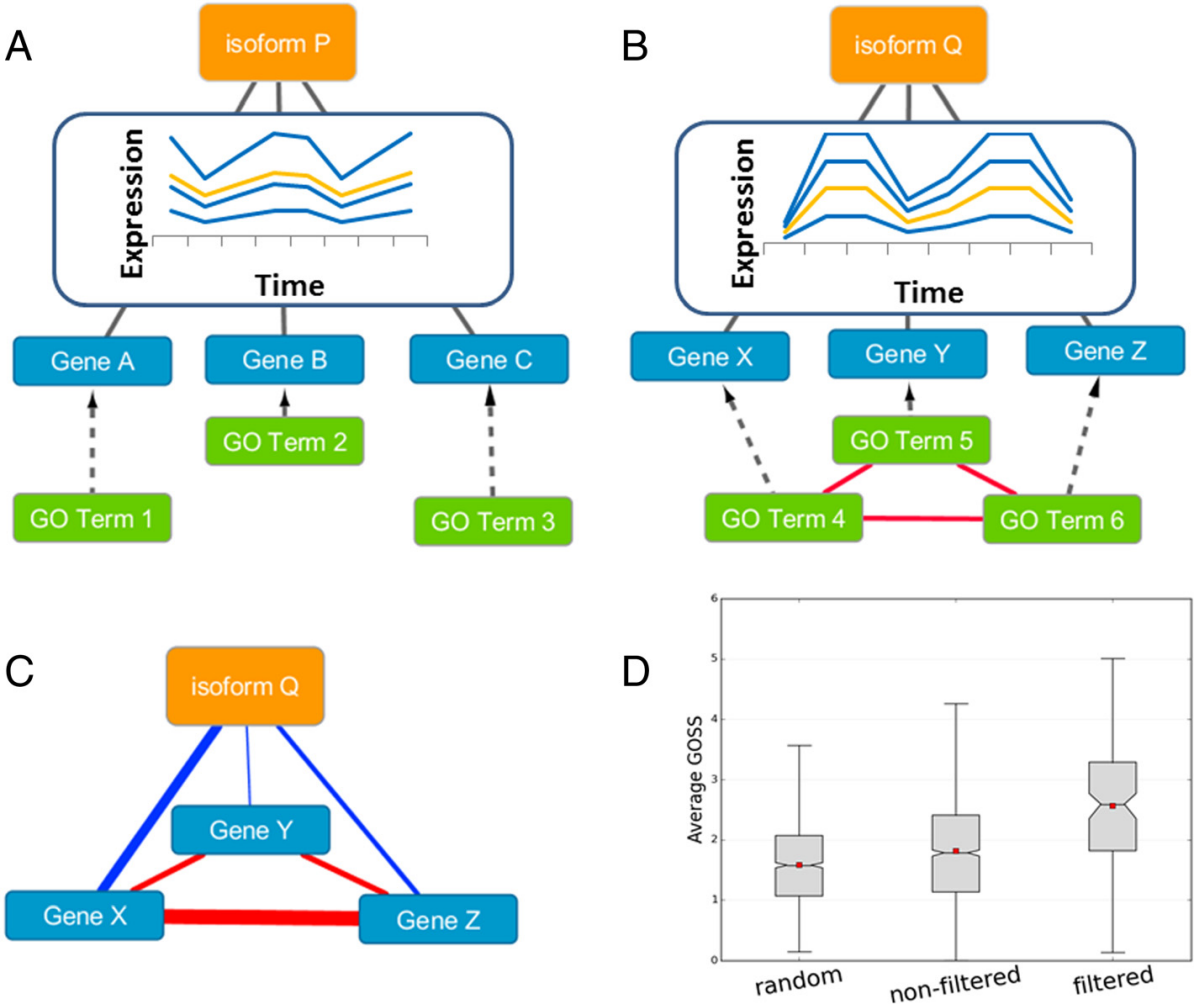
Explanation and validation of the TAPAS algorithm filtering step. For a given query isoform (orange node) a set of other genes (blue genes) are identified having correlated expression levels in their isoforms. The GO terms of these genes are compared with one another (excluding the query isoforms gene) to obtain an average GOSS score (see Methods). In example **a** isoform-P belongs to a cluster with low GOSS similarity and this cluster is discarded. In example **b** isoform-Q belongs to a cluster with high average GOSS similarity. The cluster is treated as valid and can be used to help characterise the functional neighbourhood of the query isoform. In **c** the link width represents GOSS scores between genes, the red links are used in the TAPAS filtering step. We find in the validation **d** that the average GOSS score of a cluster to the query isoforms parent gene (blue links in C) is significantly higher for filtered clusters. Note the filtering was applied only using the similarities between the non-query members of the cluster (red links in 5C). The ‘random’ plot is a control where the clusters have been generated randomly to show a background expected GOSS similarity between a cluster and the query isoform

This led us to develop our **T**ranscript **A**nnotation **P**ipeline for **A**lternative **S**plicing (TAPAS) (Fig. 6) (For details of the algorithm see Methods). In brief, the algorithm can be summarised as follows. Firstly, for a query isoform, it builds a cluster of isoforms from other genes (different to the query isoforms parent gene) whose expression patterns correlate to the query isoforms, and hence are possibly related in the same functional module. If data is available, TAPAS then builds a network between the members of the cluster using a combination of experimental protein interactions [43] and high confidence predicted functional interactions [44]. However if a cluster lacks annotated interactions we apply an optional filtering step that checks if the cluster of isoforms, have an average GO semantic similarity (GOSS) score (see methods) above a user specified cut-off (see methods), ensuring that the cluster of isoforms is both coherent in expression and function when other network data is not available.

The importance of the filtering step can be clearly seen in Fig. 6d where the functions of the isoform clusters are much more similar to the query isoform when the filtering is applied. Note that in this validation the filtering step does not use the query isoforms gene level function annotations in establishing its homogeneity, so there is no circularity involved in gaining the improvement of performance by filtering (Fig. 6c).

**Fig. 7:**
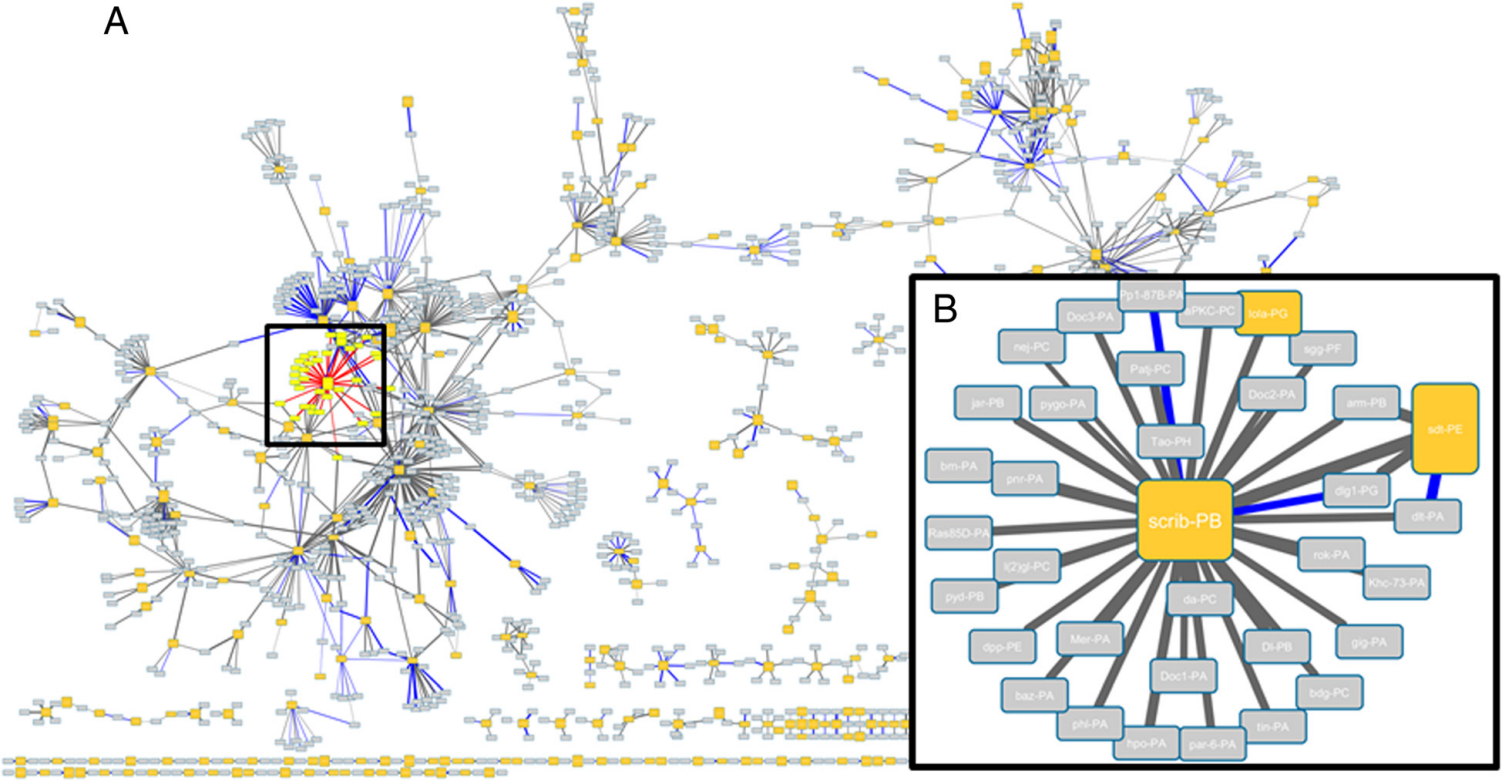
Predicted isoform gene neighbourhoods from TAPAS. **a** Output from the TAPAS algorithm for all minor isoforms from High-TS Genes. Only functional links between query isoforms (orange boxes) and co-expressed non query transcripts from different genes (grey boxes) identified by TAPAS are shown. Only links with a GOSS cut-off of > 6.0, a string score of >800 or an experimentally determined protein interaction (blue links) are shown. **b** The box shows a zoom in on two query isoforms scrb-PB and sdt-PE and their predicted functionally associated neighbours

Running the TAPAS algorithm on minor isoforms having a TS score >0.5 enables us to generate a reasonably large network of associations between isoforms from different genes (see Fig. 7). We can zoom in on a query isoform in this network, scrb-PB (Fig. 7b). This isoform is identified by TAPAS as having a similar expression pattern and functional associations with isoforms from other genes involved in processes such as adherens junction and zonular adherens assembly. The main expression peak for scrb-PB is from 2–8 hours covering developmental events such as cellularisation and gastrulation, where adherens junction and zonula adherens assembly is known to be important [45]. The primary isoform of scrb-PB is not identified as belonging to the same functional neighbourhood since it has a distinct expression profile. This allows us to infer that scrb-PB is more likely than its primary isoform to operate with these specific co-expressed partners (Fig. 7b) in adherens junction and zonula adherens assembly in early embryogenesis.

## Discussion

Generating expanded proteomes from the same gene could provide a means for generating the functional diversity needed for complex tasks such as metazoan-development. Indeed we find distinct expression patterns for a number of splice variants that suggest different isoforms can be more important at different times during development. It could be interesting to speculate how such complex patterns emerge. In one scenario, when a protein isoform first emerges we might expect it to have low expression relative to the primary isoform. This relatively low expression would allow for sequence change without the potentially disruptive effects associated with high protein abundance. Over evolutionary time scales, some of these minor splice variants could prove adaptive at different stages of development. If so the minor transcript could become expressed more highly at such time points. Eventually we could find the minor transcript contributes a substantial proportion to the overall expression profile diversity of the gene. In this work we potentially identify such isoforms with diverse transcript expression profiles using the TS score.

We find that genes with high TS scores have several characteristics consistent with them being involved in multiple functions at different times. At the sequence level, exon gains (and so potential functional gains), are more likely to be found in primary to minor isoform transitions with high TS scores. We also find that secondary protein isoforms with high TS scores tend to gain features such as ELM motifs or domains not present in the primary isoform. This demonstrates the secondary isoforms of High-TS Genes can increase the potential number of functions performed by these genes.

We show the High-TS Genes to be particularly involved in regulatory processes and supporting organism complexity through processes such as transcription factor regulation. This observation is particularly interesting given that the High-TS Genes seem to originate from a time when complex multicellular organisms were first appearing.

High-TS Genes are enriched for genes with regulatory functions. For example we find a significant enrichment in nuclear hormone receptors for High-TS Genes. This is also interesting, given that we identified LIG_NRBOX motifs as conferring functional diversity to secondary isoforms of High-TS Genes. These motifs are often found as coactivators of nuclear hormone receptors [46]. Hence we can see how the switching of isoforms in High-TS Genes could regulate, switching gene expression on and off in an integrated manner.

Enrichment in similar processes has been noted before, for a set of human genes that showed significant changes in exons between different tissues (tissue specific exon containing genes) [24]. In our analysis we use a different organism, system (development) and method (TS score) for identifying our gene sets. We also restrict our analysis to primary and secondary protein isoform events, rather than considering all transcripts. Hence there are large differences between these works. However, both papers support an important connection between the dynamics of alternative splicing and cell regulatory processes.

We also find High-TS Genes to be enriched in essential genes. We put forward two possible explanations for this. Firstly it may be advantageous for essential genes to functionally expand using alternative splicing. Alternatively High-TS Genes may carry out greater numbers of functions through their different isoforms and so it could be that knockdown of these genes equates to knockdown of multiple functions, leading to a greater chance of them having been identified as essential.

There is a great need for concerted efforts to functionally characterise different protein isoforms. Isoforms with high TS scores have expression profiles that indicate specific time points at which they may play an important role compared to the primary transcript. (see Fig. 2 for example). This feature is useful in experimental design since it provides us with the time points under which we would expect to see a phenotype on knockdown of a specific isoform.

The TS score we developed is distinct from the Pearson correlation in that both shape and magnitude are equally important in the overall score. In this regard the TS score could be useful for other applications. For example lncRNAs can negatively regulate a nearby genes expression which can be detected by anti-correlation in their expression profiles [47]. In detecting such anti-correlated gene pairs, the relative magnitude of their expression profiles could also be considered through using the TS score, to capture the strength of the effect for prioritising candidates for experimental validation.

With the TAPAS algorithm we attempted the challenging task of functionally characterising protein isoforms. The method exploits the observed distinctness in expression profile shape between primary and minor isoforms found for High-TS Genes. The TAPAS pipeline identifies clusters of isoforms from other genes with similar expression patterns to a query isoform and subsequently applies functional similarity filters on the clusters. We show this to be a key step, ensuring the cluster is functionally coherent. The co-expressed neighbours of the query isoform identified by TAPAS can help in its functional characterisation, further guiding experimental design (Fig. 7 for example).

A major caveat of this work is that all studies are carried out at the transcript level. Yet there is great uncertainty as to which transcripts go on to form stable proteins. Existing proteomics experiments suggest that only a subset of the isoforms detected at the transcript level makes it through to the proteome level [48], with a surprisingly large proportion of the proteomics-detected, alternate isoforms showing only subtle changes. As new proteomics datasets become available we will be able to test these observations further.

Another caveat is that we define the primary isoform as the most expressed isoform. Different definitions for primary isoform designation have been provided in human using the APPRIS pipeline [49]. However, these annotations are not available for fly. It would be interesting to see how often the expression based method and APPRIS like assignments agree. Clearly our analysis shows that whilst the notion of a dominant isoform generally holds for most genes, for some genes this becomes rather fuzzy, particularly where the isoform contributions become more equal.

Although the focus of this article has been on High-TS Genes, the majority of genes have low TS scores. Even if the majority of splicing events are not functional, they could provide a great deal of variety for adaptive functions to emerge. Alternative splicing has the ability to produce large changes in sequences in one go, through removal of one or more exons from a transcript for example. As already discussed, it can be expected that substantial sequence changes to a protein through processes such as alternative splicing, are more likely to cause disruptions of structure and function. Furthermore, it is known that highly expressed proteins are less likely to change their sequence [27, 50, 51]. This effect is thought to stem from deleterious changes in protein sequence (off target functions, solubility, stability) being amplified as the protein abundance increases [51]. However, if a minor protein isoform is only ever expressed at low levels and in a subset of biological contexts, it will be less likely to produce such negative consequences. Hence, even though most minor protein isoforms appear to have no functional consequence (i.e. low TS scores) they could be a major source for sequence feature innovation for an otherwise sequence constrained gene.

## Conclusions

In summary, identifying isoform specific annotation is a challenging yet critically important task. We identified a subset of genes in fly development whose secondary protein isoforms show distinct expression profiles (from the primary isoform) and also provide a large contribution to overall gene expression. These genes are not only enriched in regulatory and essential genes, but their secondary isoforms possess additional sequence features that suggest functional roles for them distinct from the primary isoform. The TS score and the TAPAS algorithm developed here provide unique methods to help in isoform target prioritisation and experimental design. Given the biological and experimental noise associated with alternative splicing, our method prioritises those splicing events that show a clear expression signal of functional importance. We show how these tools are useful for exploring the roles of alternative splicing. We anticipate the methods we develop here to be useful tools for future alternative splicing research, especially when combined with other comparative genomics and experimental datasets.

## Methods

### Isoform expression pattern based scores

For the current study we made use of FlyBase v5.45. We obtained the paired end sequence data from the fly developmental modENCODE project [22] from the European Nucleotide Archive [52] and calculated expression levels of isoforms from FlyBase 5.45 for all developmental stages. We used the most recent versions of Cufflinks2.2.1 [53], Tophat2.0.12 [54] and Bowtie2 [55]. Tophat2 was run with the following parameters (−F 0 -i 40 -p 4 -g 40 -G -T -r 200 --mate-std-dev 20).

### Identifying different splicing events

GTF files from FlyBase 5.45 were downloaded. For a given primary/minor isoform pair, an exon was deemed to be gained in the minor isoform if it did not overlap with any exons in the primary isoform whilst an intron was deemed to be retained in the minor isoform if it had an exon that fully overlapped an intron from the primary isoform.

### Identifying evolutionarily conserved exons

The set of exon co-ordinates and Conservation Index scores were downloaded from the supplementary data in the modENCODE validation paper [26]. When we plotted the distribution of scores we noticed the score distributions resembled overlapping Gaussian distributions. A conservation cut-off of 5 was used to separate the highest distribution from the low distribution and this was chosen as a cut-off for identifying conserved exons.

### Other datasets

Gene age, duplication status and essentiality data was downloaded from the OGEE resource, mapping was done using the gene identifiers. For the ‘young’ category of genes we took anything that had potentially arisen from Insecta or younger (ages 1 and 0). We treated genes of age 0 as species specific. Domain assignments were obtained from Gene3D v12.1 corresponding to Ensembl v70. So for any of the analyses in the paper using domain based analyses we used the union of protein sequences from Ensemblv70 and FlyBase 5.45.

ELMs were assigned using the ELM server, which by default applies important filters such as structural checks to ensure the ELMs are exposed and not in secondary structures of globular domains [28]. Additionally we applied the taxonomic range filter to remove ELMs not related to *D. melanogaster*. Sequence specific DNA binding domains were obtained from the DBD [38] resource. Sequences with classical signalling domains were obtained from the SMART website [39].

### Bias removal for essential gene enrichment analysis

We developed an approach to control for any potential subtle biases associated with potential differences in gene expression patterns and levels. For each High-TS gene, we found the multi protein gene (that was not a High-TS gene) with the closest matching gene expression profile as measured by the Euclidean distance. This was done iteratively such that after a multi protein gene was paired with a High-TS gene it could not be paired with another High-TS gene. We subsequently re-checked for enrichments using this paired set where the gene expression profiles were highly similar even though the isoform expression patterns could be very different.

To control for biases in singleton gene enrichment for High-TS Genes, the essential gene enrichment was repeated using only singleton genes. To control for biases in signalling and DBD gene enrichment the essential gene enrichment was repeated using multi protein genes with signalling and DBD domain containing genes removed. To control for biases in gene age we only included genes from gene ages Metazoa or older.

### Enrichment analysis

The Fisher’s exact test was implemented using the fisher python package v0.14) for testing gene set enrichments. The Mann–Whitney rank test was implemented using the scipy.stats package. For both Fisher and Mann–Whitney rank test all p-values given are one sided.

Functional enrichment was carried out using the gene association GO annotation file from FlyBase. We filtered ND and NOT evidence code annotations. For correcting for multiple tests we used the Benjamini-Hochberg method. GO terms with less than 10 gene annotations in the background set were not considered for testing.

### FunFam assignments

Functional families, (FunFams), are clusters of functionally related domain sequences, built by agglomerative clustering of Gene3D [29] domain sequences based on the similarity of sequence clusters measured by profile–profile comparisons. Gene3D is a resource providing all predicted domain sequences assigned to superfamilies in the CATH structural classification [17]. The method has recently been updated and makes uses of specific, function determining, residues in multiple sequence alignments of the family to determine when to stop merging clusters [17]. FunFam assignments for Drosophila were downloaded from the Gene3D v12 website.

### Fun-L ranking

Fun-L provides a means of identifying functionally related genes using gene networks. FunL converts protein protein association networks into similarity matrices that can then be used for identifying related genes [43]. We used this method to identify a small number of genes predicted to be highly associated to the set of nuclear hormone receptors having high TS scores, to help produce a more connected network*.* Fun-L is relevant to Fig. 4 in the results.

### GOSS Score

A network of GO term similarities was generated using the Resnik GO semantic similarity (GOSS) score [56]. The GOSS score has been widely used previously and provides a simple means of measuring similarity between GO terms. The GOSS score between any two GO terms is inversely related to the number of genes assigned to the common parent of the GO terms, such that if the shared parent GO term is assigned many genes (i.e. the term is non-specific) then the GOSS score linking the GO terms is relatively low. The usual filters were also applied to remove annotations with Not Determined (ND) and terms with negations (NOT) indicating the GO term was not assigned to the gene. GOSS similarities between two genes were assigned using the maximum GOSS score from their genes GO annotations.

### TAPAS algorithm

The TAPAS algorithm proceeds as follows. For a query isoform, it builds a cluster of isoforms from other genes (different to the query isoforms gene) whose expression patterns correlate to the query isoforms (By default Pearson similarity > 0.7), and hence could be related in the same functional module. From these proteins, a network of proteins interacting with the query isoform is built to identify clear functional links between the query isoform and other members of the cluster. The links are made up of experimental protein interactions, high confidence STRING predictions (score > 800) and highly specific GO semantic similarity links (GOSS) (default is GOSS score >6).

In cases where no functional links can be established using STRING, known interaction or high GO similarity (GOSS > 6), TAPAS applies a further filtering step, that checks if the cluster of the closest 20 isoforms with GO terms, have an average GO semantic similarity (GOSS) score (see below) above a given cut-off (as default we chose a GOSS score of 2.5 since this is far above the random background score expected). Only GO terms from the biological process branch of the Gene Ontology were used since we would expect similarities in GO terms from this branch to be better reflected in expression profile similarities than other branches (e.g. Molecular Function).

### Availability

The TAPAS code and predictions are available for download for download (ftp://ftp.biochem.ucl.ac.uk/pub/gene3d_data/CURRENT_RELEASE/TAPAS).

## Additional file

Additional file 1: Figure S1. Example of a secondary isoform with a low TS score when compared to the primary isoform. Figure S2: Plot of average correlation (Pearson), and expression ratio for various TS score bins. The expression ratio is the average secondary isoform expression/average primary isoform expression. Figure S3: Correlation plot between various scores derived from comparing the primary and secondary isoform expression profiles. The grid of colours and numbers show correlations between any two scores. Note ‘Pearson * -1’ was used to turn into a distance measure. The ‘Expression Ratio’ is the ratio of the average secondary isoform expression to the average primary isoform expression. The TS score shows high correlation with both the Expression ratio and Pearson * -1 indicating it is the most suitable score for the study (we multiply Pearson by -1 for visualisation reasons, so it increases with distance, like the Euclidean etc.). Jensen-Shannon Divergence is abbreviated as JSD. For JSD-2 the two input distributions for JSD are changed to the primary isoform expression profile and the primary expression profile + secondary expression profile. Figure S4: Histogram of TS scores.
