# Supplementary material for: Identifying and characterising key alternative splicing events in Drosophila development
Source: BMC Genomics. 2015 Aug 16;16(1):608. doi: 10.1186/s12864-015-1674-2 (PMC4537583; doi:10.1186/s12864-015-1674-2)
Supplement: Additional file 1: Figure S1. — Example of a secondary isoform with a low TS score when compared to the primary isoform. Figure S2: Plot of average correlation (Pearson), and expression ratio for various TS score bins. The expression ratio is the average secondary isoform expression/average primary isoform expression. Figure S3: Correlation plot between various scores derived from comparing the primary and secondary isoform expression profiles. The grid of colours and numbers show correlations between any two scores. Note ‘Pearson * -1’ was used to turn into a distance measure. The ‘Expression Ratio’ is the ratio of the average secondary isoform expression to the average primary isoform expression. The TS score shows high correlation with both the Expression ratio and Pearson * -1 indicating it is the most suitable score for the study (we multiply Pearson by -1 for visualisation reasons, so it increases with distance, like the Euclidean etc.). Jensen-Shannon Divergence is abbreviated as JSD. For JSD-2 the two input distributions for JSD are changed to the primary isoform expression profile and the primary expression profile + secondary expression profile. Figure S4: Histogram of TS scores. [file 12864_2015_1674_MOESM1_ESM.pdf]

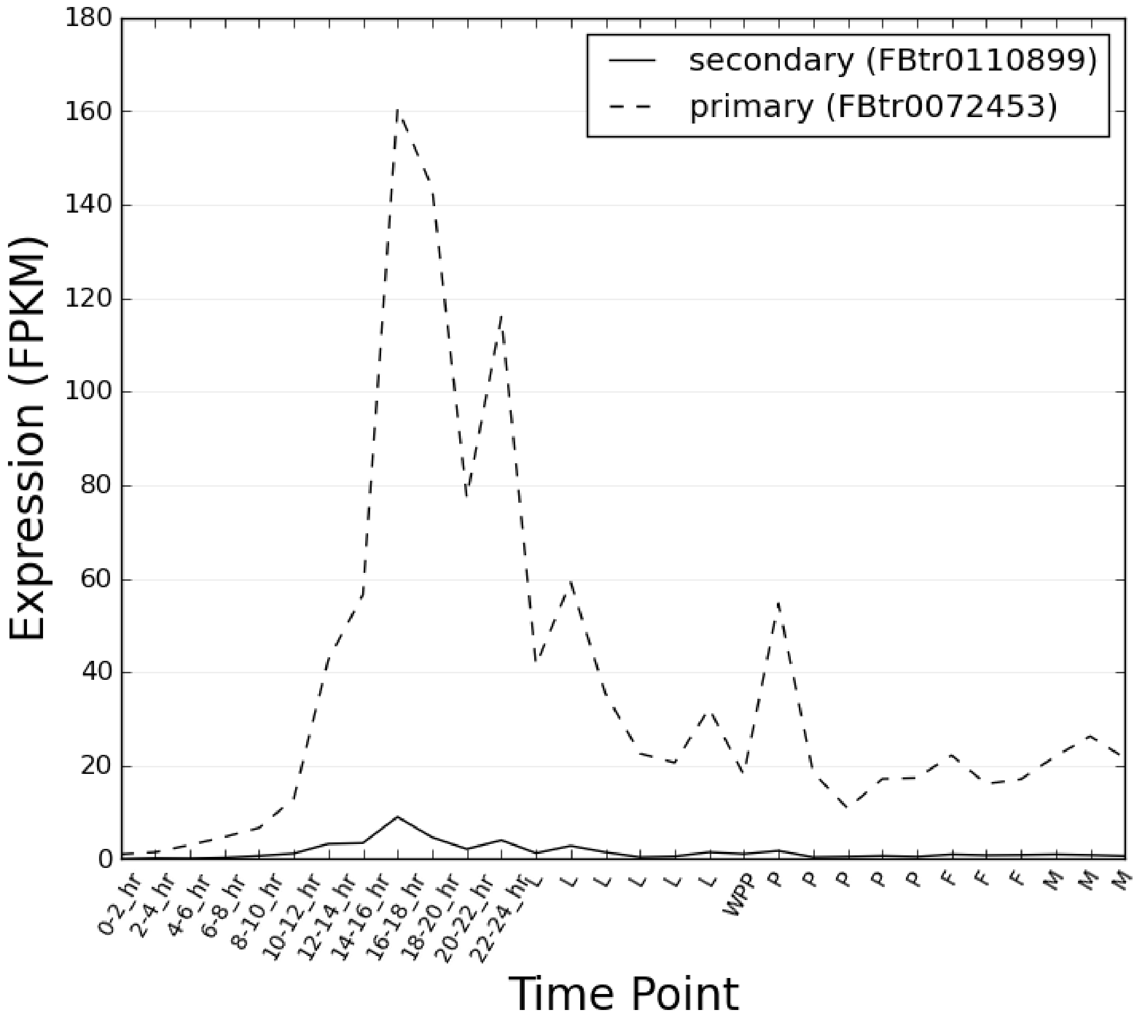

Supp. Figure 1: Example of a secondary isoform with a low TS score when compared to the primary isoform.

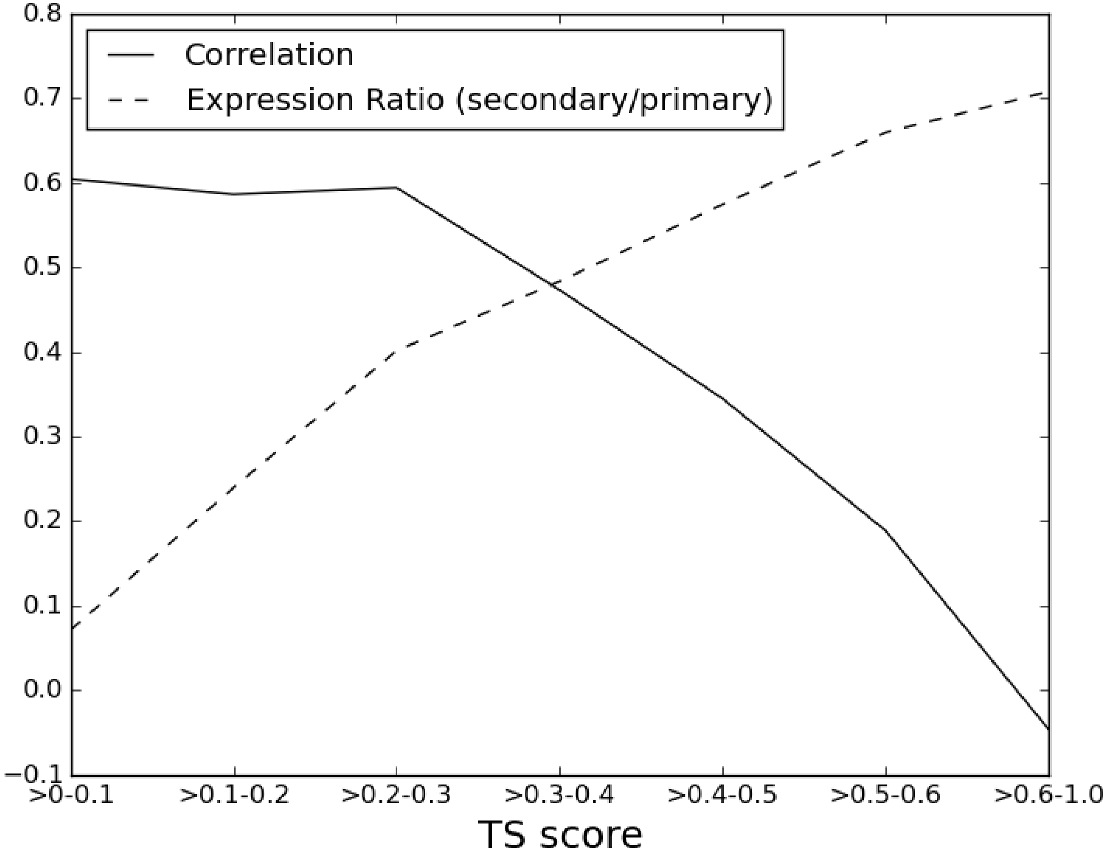

Supp. Figure 2: Plot of average correlation (Pearson), and expression ratio for various TS score bins. The expression ratio is the average secondary isoform expression / average primary isoform expression .

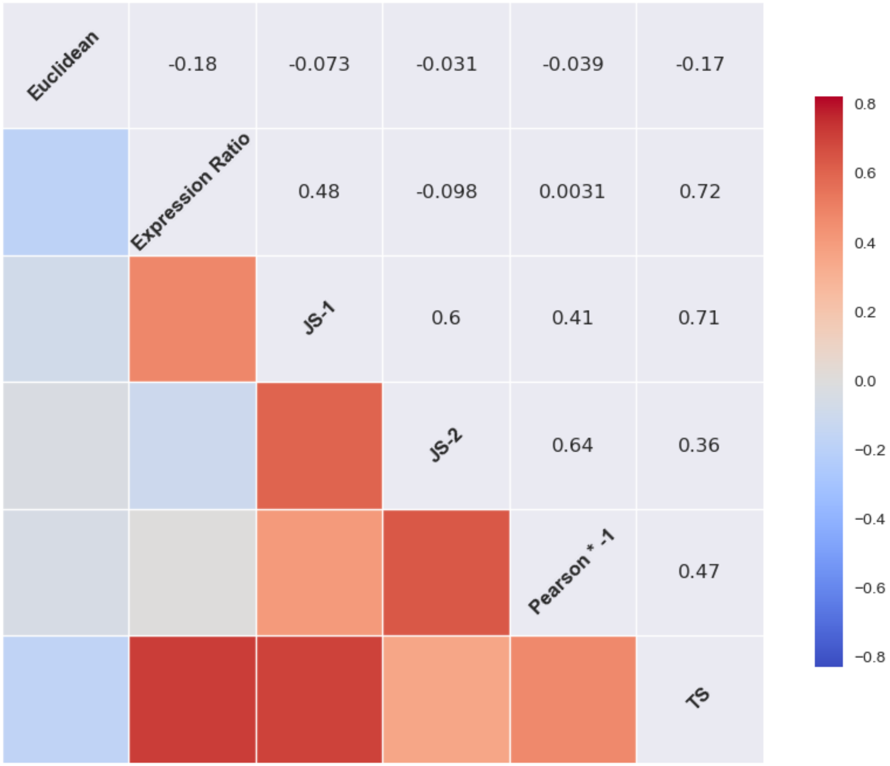

*Supp. Figure 3: Correlation plot between various scores derived from comparing the primary and secondary isoform expression profiles . The grid of colours and numbers show correlations between any two scores. Note 'Pearson * -1' was used to turn into a distance measure. The 'Expression Ratio' is the ratio of the average secondary isoform expression to the average primary isoform expression. The TS score shows high correlation with both the Expression ratio and Pearson * -1 indicating it is the most suitable score for the study (we multiply Pearson by -1 for visualisation reasons, so it increases with distance, like the Euclidean etc.). Jensen-Shannon Divergence is abbreviated as JSD. For JSD-2 the two input distributions for JSD are changed to the primary isoform expression profile and the primary expression profile + secondary expression profile.*

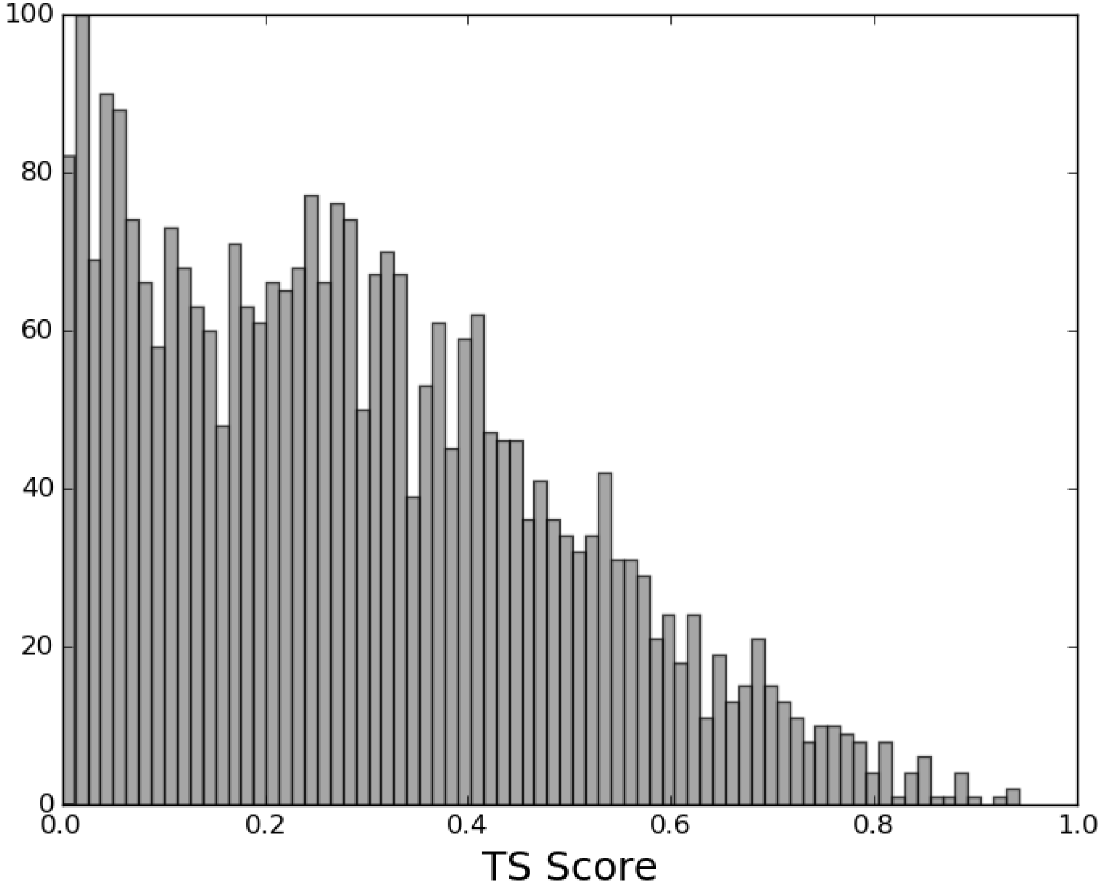

Supp. Figure. 4: Histogram of TS scores.
